# Paternal care plasticity: males care more for early- than late-developing embryos in an arboreal breeding treefrog

**DOI:** 10.1186/s12983-024-00537-z

**Published:** 2024-06-19

**Authors:** Yuan-Cheng Cheng, Cai-Han Xie, Yu-Chen Chen, Nien-Tse Fuh, Ming-Feng Chuang, Yeong-Choy Kam

**Affiliations:** 1 Department of Life Science, Tunghai University, Taichung, 407224 Taiwan; 2 Department of Life Sciences and Research Center for Global Change Biology, National Chung Hsing University, Taichung, 402202 Taiwan

**Keywords:** Parental care, Paternal care, Harm to offspring hypothesis, Offspring vulnerability hypothesis, Plasticity, Phytotelm-breeding, Treefrog

## Abstract

**Background:**

Parental care benefits offspring but comes with costs. To optimize the trade-off of costs and benefits, parents should adjust care based on intrinsic and/or extrinsic conditions. The harm to offspring hypothesis suggests that parents should invest more in younger offspring than older offspring because younger offspring are more vulnerable. However, this hypothesis has rarely been comprehensively tested, as many studies only reveal an inverse correlation between parental care and offspring age, without directly testing the effects of offspring age on their vulnerability. To test this hypothesis, we studied *Kurixalus eiffingeri*, an arboreal treefrog with paternal care. We first performed a field survey by monitoring paternal care during embryonic development. Subsequently, we conducted a field experiment to assess the prevalence of egg predators (a semi-slug, *Parmarion martensi*) and the plasticity of male care. Finally, we conducted a laboratory experiment to assess how embryo age affects predation by *P. martensi*.

**Results:**

Our results showed that (1) male attendance and brooding frequency affected embryo survival, and (2) males attended and brooded eggs more frequently in the early stage than in the late stage. The experimental results showed that (3) males increased attendance frequency when the predators were present, and (4) the embryonic predation by the semi-slug during the early was significantly higher than in the late stage.

**Conclusions:**

Our findings highlight the importance of paternal care to embryo survival, and the care behavior is plastic. Moreover, our results provide evidence consistent with the predictions of the harm to offspring hypothesis, as males tend to care more for younger offspring which are more vulnerable.

**Supplementary Information:**

The online version contains supplementary material available at 10.1186/s12983-024-00537-z.

## Background

Selection favors phenotypes, including behaviors, that can enhance the gross fitness of animals. Parental care can provide benefits to offspring through various forms to increase offspring fitness and the parents’ reproductive success [1]. However, parents often incur potential costs, including survival and reproductive costs, such as increased predation risk [2], reduced food acquisition [3], and delayed time to the next mating [4]. Theoretically, parental care can evolve when the benefits of providing care outweigh the costs [1, 5]. Nevertheless, the costs and benefits of parental care vary with intrinsic factors (e.g., parental age and condition) and/or extrinsic factors (e.g., temperature, moisture, predator risk, offspring relatedness, brood size, and quality) [6–9]. Therefore, parents should adjust their investments based on both intrinsic and extrinsic conditions to maximize the benefits and minimize the costs [1, 6, 9].

Research across various taxa has shown that changes in extrinsic conditions can strongly influence parental care decisions (e.g., birds [10], snakes [11], lizards [12], and frogs [7]). In frogs, one common form of parental care is egg-guarding behavior [13], which includes attendance and brooding. These behaviors can provide benefits to the eggs [14], such as protecting them from predators [15], removing poorly developed or fungus-infected eggs, maintaining optimal humidity [16], or transferring antimicrobial substances [17]. Some frog species with egg-guarding behavior exhibit a fixed, high frequency (almost full-time) of parental care. For example, in *Chiromantis hansenae*, the mother cares for the eggs throughout the entire egg phase, and experimental removal of parental care results in complete egg mortality [15]. However, performing care of eggs imposes high costs on parents, including exposure to higher predation risk, energy loss, reduced food intake, and missed mating opportunities [18–20]. Therefore, studies on some frog species have found that parents adjust their parental care in response to environmental changes to reduce costs (e.g., [7, 8]).

The age of offspring is an external factor that can influence parental care. Parents can adjust their investment in parental care based on their offspring’s developmental stages (i.e., age) [1, 21]. Two hypotheses regarding the effect of offspring age on parental care have been proposed: the parental investment theory (or offspring value hypothesis) suggests that parents should provide more care as offspring value increases, expecting parents to invest more in older offspring because they represent more accumulated parental investment and therefore higher value [1, 22, 23]. This prediction has been confirmed in many bird studies [24, 25]. Alternatively, the harm to offspring hypothesis (or offspring vulnerability hypothesis) suggests that parents should provide more care when offspring are more vulnerable. Therefore, it expects that parents should care for younger offspring more because they are more vulnerable, while older offspring, being less vulnerable (e.g., better able to resist predators), can still survive without or with less parental care [21, 26]. For example, in parasitoid wasps (*Goniozus nephantidis*) that exhibit infanticide behavior, females defend younger larvae more than older ones when facing intruders (predators) of the same species [27]. Some other studies have also found correlations between parental care and offspring age that are consistent with the harm to offspring hypothesis (e.g., insects: [27]; birds: [21]; mammals: [28]). In these studies, parental care was observed to be higher in the early stages and lower in the later stages. Whether younger offspring are more vulnerable, however, has rarely been directly tested.

The Eiffinger’s treefrog (*Kurixalus eiffingeri*) is a small treefrog that exhibits parental care and is commonly distributed in montane-clouded forests of Taiwan. They typically breed in phytotelmata, such as tree holes and bamboo stumps, where males guard the eggs during the egg phase, and females feed the tadpoles by laying unfertilized eggs after hatching [29]. Previous studies on *K. eiffingeri* found that males generally exhibit less than 30% attendance during the egg stage [30, 31], and males do not guard the eggs throughout the entire period: attendance frequency is higher in the early and lower in the late developmental stage [30, 31]. The primary cause of hatching failure in *K. eiffingeri* was predation by an invasive species, the semi-slug *Parmarion martensi* [32]. Guarding by male frogs can effectively reduce egg (and embryo) predation [32]. Meanwhile, male *K. eiffingeri* can adjust their care behavior accordingly in response to the variation of nest value (i.e., clutch size) and predation risks [33]. Therefore, *K. eiffingeri* provides an ideal species for testing the harm to offspring hypothesis. According to this hypothesis, since late-stage embryos have more fully developed muscle tissue compared to early-stage embryos, which helps hinder predators from consuming them, we expect males to exhibit more care during the early stages of embryo development when the embryos have weaker defenses against predators, and decrease care frequency in the later stages when the embryos have better predator resistance [21, 26].

This study aims to investigate the harm to offspring hypothesis using *K. eiffingeri*. First, we conducted a field survey to monitor male guarding behavior and the survival of embryos, confirming (1) the effect of paternal care on embryonic survival and (2) differences in male guarding (attendance and brooding) frequency between early and late embryonic development stages. Subsequently, we conducted field experiments using *P. martensi* as a predator to test (3) whether its presence would cause males to alter their guarding (attendance and brooding) frequency. Finally, through a laboratory experiment using *P. martensi*, we examined (4) whether embryos at different developmental stages exhibit different resistance to predation. The (1) and (3) in this study were to confirm the importance of paternal care to embryo survival and the plasticity of parental care. Additionally, (2) and (4) served to test the harm to offspring hypothesis, as they investigated the relationship between male investment in paternal care and offspring age, as well as the relationship between offspring age and vulnerability.

## Methods

### Study time and site

This research was conducted from July to August of 2020 and July to August of 2021. We conducted field experiments and collected animals from two bamboo forests (23°68′85″ N, 120°79′10″ E and 23°68′90″ N, 120°79′10″ E) in Chitou, Nantou County, Taiwan. The bamboo forests consisted of *Phyllostachys edulis* and *Sinocalamus latiflorus*, which are periodically cut for commercial purposes, and the internodes left after bamboo cutting formed stumps that accumulated rainwater. *K. eiffingeri* utilized these bamboo stumps as breeding sites. Chitou is situated at an elevation of approximately 1016 m, with an average annual temperature of 17 °C and an annual rainfall of about 3000 mm, with rainfall concentrated mainly from February to September [29].

### Study I: field survey

#### Sampling of clutches and males

The observation aimed to examine the effects of paternal care on embryo survival and to compare the differences in paternal care between early and late embryonic development stages. From July to August 2020, we searched in the study area for newly laid eggs of *K. eiffingeri* (1 to 2 days old). We recorded the clutch size and captured the males (usually resting at the bottom of bamboo stumps during the day and perching or guarding the eggs at night). We measured the males’ snout-vent length (SVL), marked them by toe clipping [34, 35], and released them back into the bamboo stumps. We also measured the bamboo stump’s characteristics, including stump inner diameter, height, depth, and water depth (cf. [36]).

#### Monitoring of males and eggs

After marking males, we set up infrared cameras (Ereagle E1B Waterproof Trail Camera) near the bamboo stumps. The cameras were positioned 50 cm away from the egg-laying site and were set to take one photo per minute inside the bamboo stumps every day until all eggs hatched or died. During the monitoring, we checked focal clutches daily between 3 and 5 pm, and recorded the egg number. After all eggs were hatched, we calculated the hatching rate for each clutch.

#### Quantification of paternal care and embryo mortality

By using photos from monitoring, we quantified the frequency of paternal care behaviors. Attendance and brooding were the focal egg-guarding behaviors in this study. Each of these behaviors persisted for more than 1 min each time it was performed. Therefore, taking a photo every minute ensured that almost all instances of parental care were recorded. For each clutch, we defined the *total recordings* as the number of photos taken from the beginning until the first tadpole hatched (or until all eggs died if no tadpole hatched). The number of photos capturing males engaging in attendance or brooding represented the care number. First, we calculated the *attendance frequency* as “the number of photos of a male showing up at the spawning site / total recordings” and *brooding frequency* as “the number of photos of a male physically in contact with the eggs / total recordings”. In rare cases, males stayed at the inner wall positions (which referred to attendance), which were blind spots for the camera. In these cases, we determined if a male frog had stayed inside the bamboo stump by examining photos taken before and after. Our camera angles were always positioned to capture the eggs, ensuring no blind spots for recording males’ brooding behavior. Second, we calculated the *average daily embryo mortality rate* as: $\frac{\text{Total number of embryo deaths}}{\text{Total amount of embryos in a clutch}}\div \text{ number of days until no more embryos died}$. Third, to compare paternal care during the early and late stages of embryonic development, we calculated the attendance and brooding frequencies in the early stage and in the late stage. We categorized the development of the embryo according to the Gosner stage (hereafter, G-stage) [37]. The early stage refers to the embryo before the 18^th^ G-stage, while the late-stage group refers to the embryo at and after the 18^th^ G-stage.

### Study II: field manipulative experiment

#### Experimental design and procedure

**Fig. 1:**
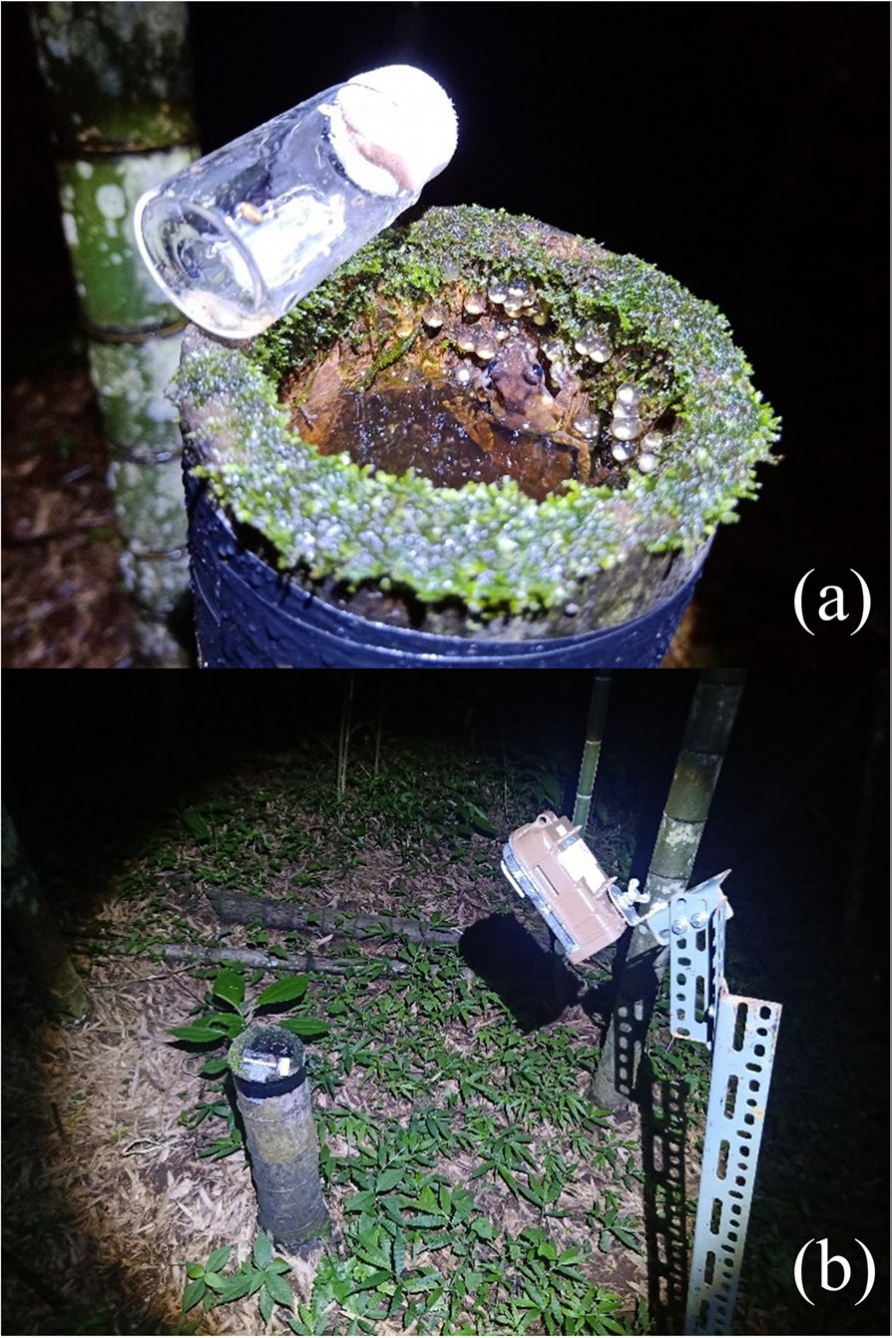
The setup of the manipulative experiment in the field. **a** In the experimental group, a semi-slug in a transparent glass jar was placed on the upper rim of the bamboo stump. **b** During the experimental period, the cameras were positioned 50 cm away from the egg-laying site to monitor the eggs and males

This experiment aimed to investigate whether the presence of embryo predators would affect paternal care. From July to August 2021, we searched for newly laid eggs (remaining in the early stage) with a male presence. We measured males’ SVL, marked them by toe clipping, and released them back into the bamboo stumps. We also measured the bamboo stump’s inner diameter, height, depth, and water depth [36]. We recorded the clutch size and randomly assigned the clutches into control and experimental groups. A transparent glass jar with a white plastic lid was placed on the upper rim of the bamboo stump (Fig. 1a). The glass jar was attached to the transparent plastic sheet using hot glue to secure its position and orientation. Additionally, we used insulating tape to fix the glass jar on the upper edge of the bamboo stump. A circular hole was drilled in the plastic lid, and a mesh was attached to the lid to disperse the odor inside the jar, allowing the resident male to recognize the species inside the jar. We maintained the humidity by putting cotton in the jar. We also put some moss in the jars to provide adequate food for predators. In the experimental group’s jar, a representative egg predator, *P. martensi*, was introduced to simulate increased egg predation pressure. In the control group’s jar, no predators were placed except for the cotton and moss. Finally, we measured the site characteristics of the bamboo stumps.

#### Monitoring of males and calculation of paternal care

After setting up the experiment, we installed infrared cameras as we did in study 1 and monitored until all eggs either hatched or died (Fig. 1b). We checked the clutches daily between 7 pm and 12 am, recorded the number of embryo deaths, and noted whether the males left the egg clutches. Then, we used the same method as study I to calculate male attendance and brooding frequencies for each clutch.

### Study III: laboratory manipulative experiment

#### Experimental design

This experiment aimed to examine the effect of embryonic development on predation. The experimental treatments were divided into early-stage and late-stage embryonic development groups. We collected the early-stage embryos from the wild and incubated them in the laboratory until they reached the late stage. We then used *P. martensi* as the predator to examine the predation on embryos in different developmental stages.

#### Experimental procedure

From July to August 2020, we collected the eggs from the field within 1 day of oviposition and brought them back to the laboratory. We randomly allocated these eggs into groups, each containing five eggs. Using the stickiness of *K. eiffingeri* egg jelly, we adhered eggs to filter paper to carry the eggs accordingly. Each set consisted of 5 eggs adhered to filter paper, which were subsequently attached to the inner wall of plastic containers, mimicking the natural attachment of eggs to bamboo stumps in the wild. Before the experiment, we collected *P. martensi* from the wild and starved them for 2 days. After 2 days of starvation, we measured their weight by an electronic scale (to the nearest 0.01 g). One *P. martensi* was then placed in each container during the experimental period, which was conducted from 6 pm to 6 am the next day using a camera in night vision mode (Sony HDR-CX900). We conducted 20 independent replicates for each treatment group. Then, we calculated the percentage of eggs consumed by *P. martensi*.

### Statistical analysis

All statistical analyses were performed using JMP Pro 14 (SAS Institute Inc., Cary, NC, USA), with a significance level of α = 0.05.

Due to the non-normal distribution of all the data, we used nonparametric statistics for all analyses. In study I, the Wilcoxon signed-rank test was used to examine differences in the average daily embryo mortality rate between groups with and without male attendance and brooding. In addition, the Wilcoxon matched-pairs signed-rank test was used to assess whether there were differences in male attendance and brooding frequencies between the early and late embryonic stages. In the field experiment (study II), the Wilcoxon signed-rank test was used to detect differences in attendance and brooding frequencies between the control and experimental groups. Finally (study III), the Wilcoxon signed-rank test was used to assess whether the developmental stage of the embryos affected the predation percentage by *P. martensi*.

## Results

### Study I: field survey

We monitored a total of 32 clutches in this study, each with a male, presumably the father, who remained with the clutch. All 32 clutches had complete camera monitoring records, from the initial laying of the eggs to complete hatching or death.

#### Daily mortality rate of clutches with and without paternal care

**Fig. 2:**
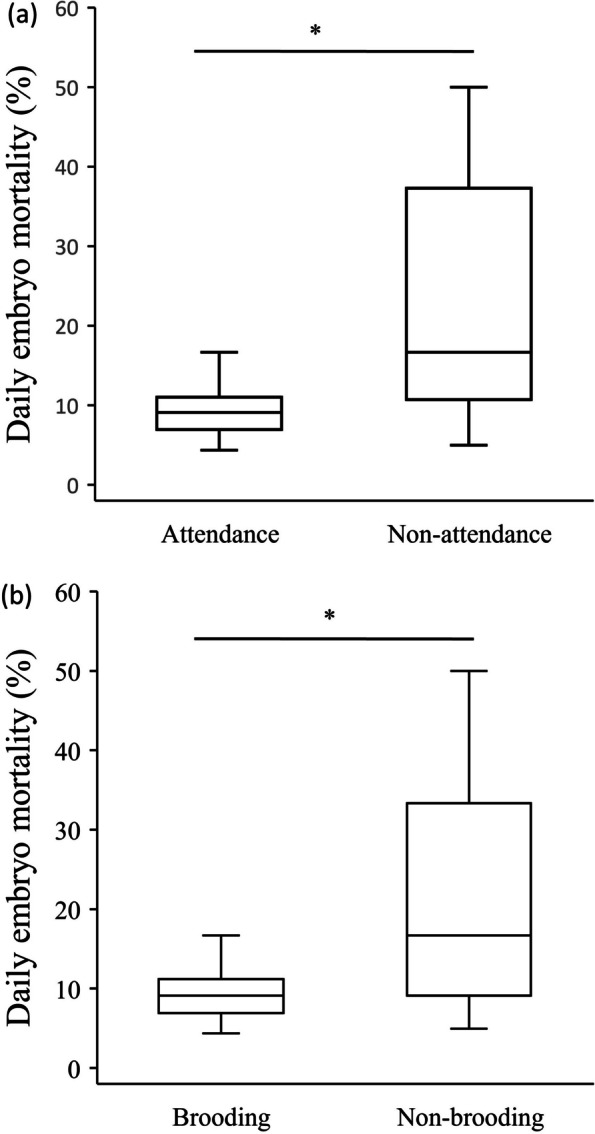
Comparisons of the daily embryo mortality in clutches between **a** the presence of attending males (Attendance) and the absence of males (Non-attendance), and between **b** the presence of brooding males (Brooding) and the absence of males (Non-brooding). The line inside the box is the median, and the bottom and top of the box refer to the 1st and 3rd quartiles. *: *p*-values less than 0.05

Among the 32 clutches, 22 of them had males engaging in attendance. Clutches with male attendance showed a significantly lower daily mortality rate than those without male attendance (Wilcoxon signed-rank test, *Z* = 2.4, *p* = 0.017, n: attendance = 22, non-attendance = 10, Fig. 2a). For the 32 clutches, 21 of them had males engaging in brooding. Clutches with male brooding exhibited a significantly lower daily mortality rate than those without male brooding (Wilcoxon signed-rank test, *Z* = 2.23, *p* = 0.026, n: brood = 21, non-brood = 11, Fig. 2b). Male SVL and bamboo stump characteristics did not correlate in a statistically significant manner with daily mortality rate (Spearman correlation, Additional file 1-Table S1).

#### Paternal care in early and late embryonic stage

**Fig. 3:**
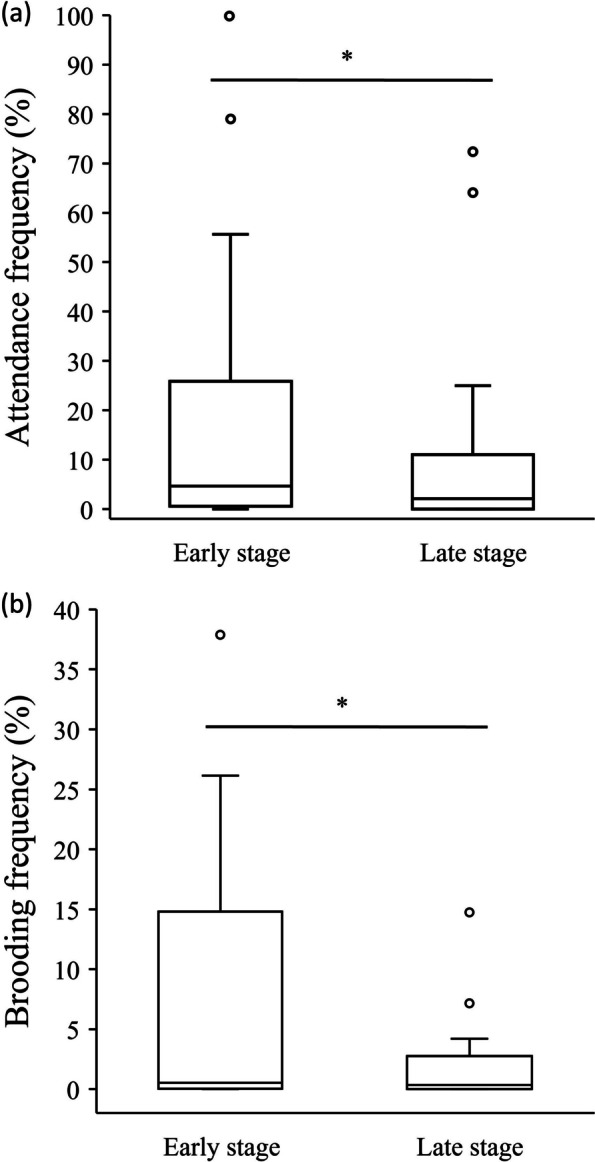
Comparisons of the males’ **a** attendance frequency and **b** brooding frequency of the clutches between early and late stages of embryo development. The line inside the box is the median, and the bottom and top of the box refer to the 1st and 3rd quartiles. Open circles represent the outliers. *: *p*-values less than 0.05

Among the 22 clutches with paternal care (attendance or brooding), excluding those that did not successfully hatch (six clutches), we analyzed the differences in attendance and brooding frequencies between embryos’ early and late stages. The results showed that the male’s attendance frequency was significantly higher in the early stages than in the later stages (Wilcoxon matched-pairs signed-rank test, *W* = -39.0, *p* = 0.044, n = 16, Fig. 3a). Similarly, the brooding frequency of males was significantly higher in the early stages than the later stages (Wilcoxon matched-pairs signed-rank test, *W* = -42.0, *p* = 0.029, n = 16, Fig. 3b).

### Study II: field manipulative experiment

We observed 15 clutches in total, with eight for the control group and seven for the experimental group.

#### Differences in paternal care between experimental and control groups

**Fig. 4:**
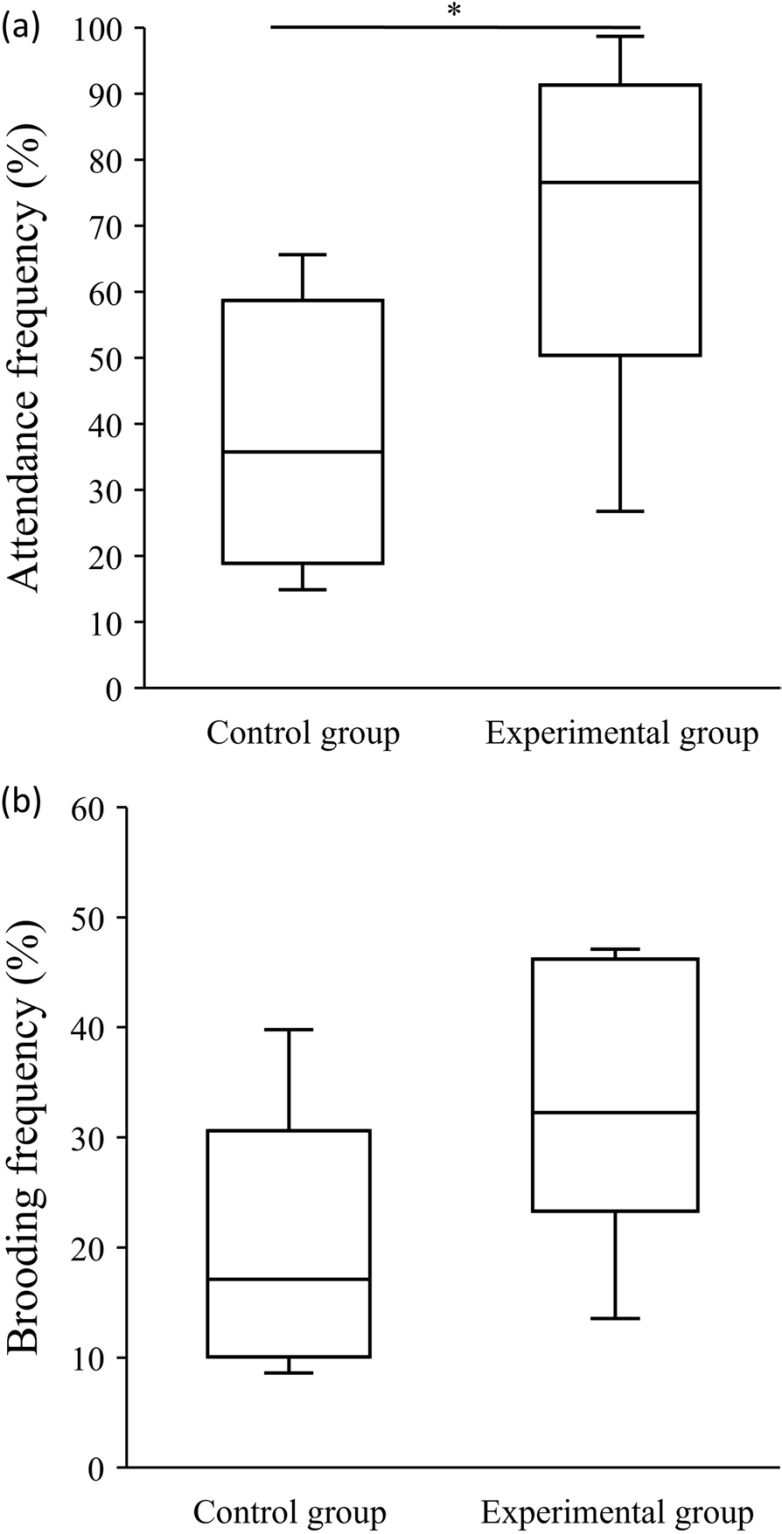
Comparison of the **a** attendance frequency and **b** brooding frequency of males between the control group and the experimental group. In the control group, there were no *P. martens* in the glass containers, while in the experimental group, one *P. martens* was placed in the glass container. The line inside the box is the median, and the bottom and top of the box refer to the 1st and 3rd quartiles. *: *p*-values less than 0.05

There was a significant difference in attendance frequency between two groups, with the experimental group (with predatory stimulus) of males exhibiting a higher attendance frequency than the control group (without predatory stimulus) (Wilcoxon signed-rank test, *Z* = 2.3, *p* = 0.024, Fig. 4a). However, there was no significant difference in brooding frequency between the groups (Wilcoxon signed-rank test, *Z* = 1.8, *p* = 0.073, Fig. 4b). There were no significant differences in male SVL and bamboo stump characteristics between the two treatment groups (Wilcoxon signed-rank test, Additional file 1-Table S2).

### Study III: laboratory manipulative experiment

**Fig. 5:**
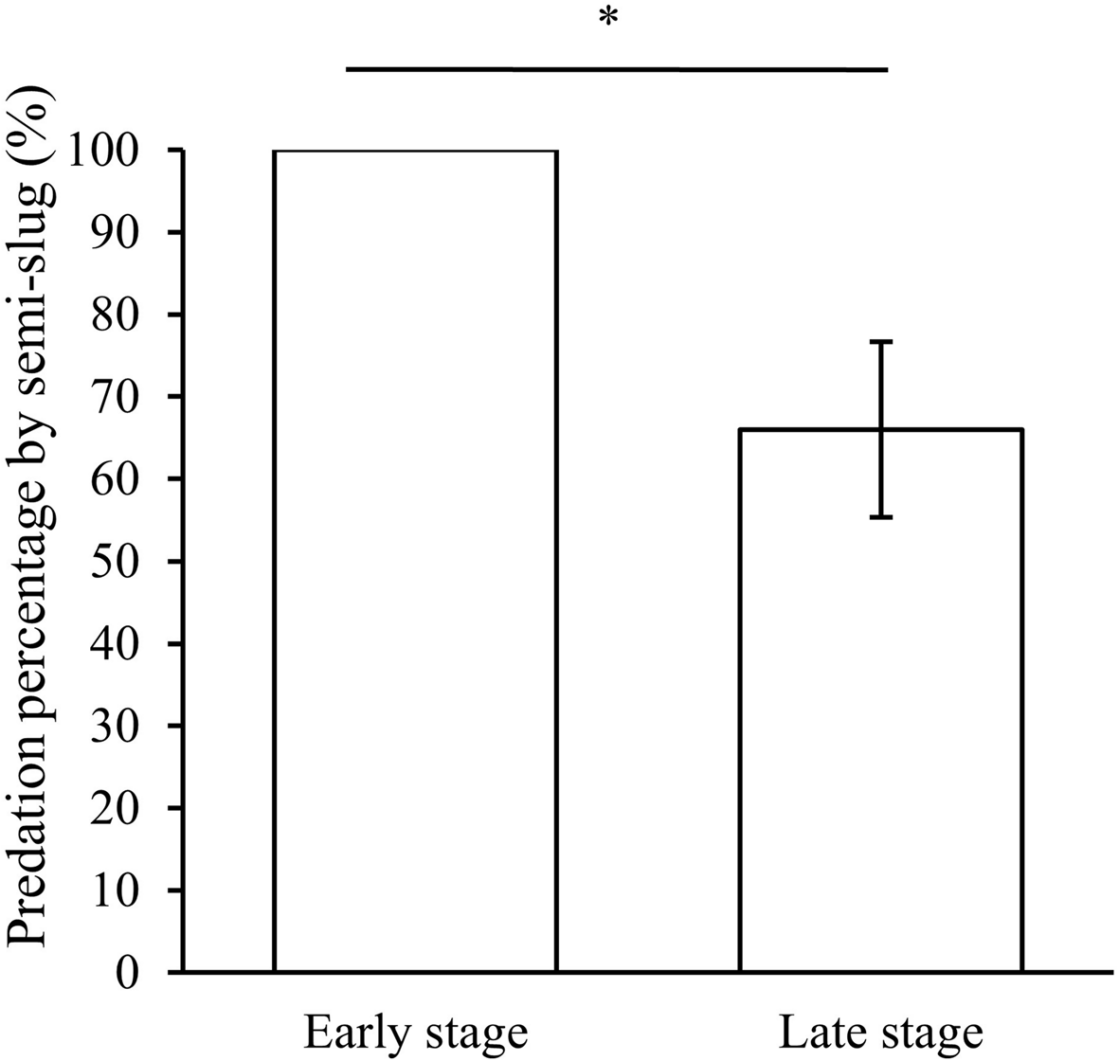
Comparisons of predation percentage by *P. martens* on embryos between early and late stages. The lengths of the bars represent means. The vertical bar is the standard error. *: *p*-values less than 0.05

The predation percentages by *P. martensi* were significantly higher in the early stage of embryos than in the later stage of embryos (Wilcoxon signed-rank test, *Z* = -2.8, *p* = 0.004, n: early stage = 20, late stage = 20, Fig. 5). The body weight of *P. martensi* did not correlate in a statistically significant manner with the percentage of predation (Spearman correlation, Additional file 1-Table S3).

## Discussion

This study aimed to investigate the harm to offspring hypothesis by examining the relationship between investment in paternal care and offspring age (embryo developmental stage) in *K. eiffingeri*, and whether offspring vulnerability is related to their age. Firstly, results showed that male egg-guarding behavior (attendance and brooding) significantly reduced embryo mortality, and males increased attendance frequency in response to the experimentally elevated predation risk on embryos. These findings confirm the crucial role of paternal care for embryo survival and demonstrate that males can adjust parental care based on predation risk. Additionally, males had higher guarding frequency in the early than late stages of embryos, and the latter exhibited better predator resistance. These results are consistent with the predictions of the harm to offspring rather than the offspring value hypotheses, as males tend to care more for younger offspring which are more vulnerable.

### The importance of paternal care and its plasticity

Male attendance and brooding are associated with lower mortality, indicating the crucial role of male care on embryo survival, consistent with previous studies on *K. eiffingeri* [31]. Parental care has been shown to influence egg mortality rates in various frog species [14]. Parents can behave in different ways to enhance egg survival, such as moistening eggs [16, 38], defending against predators [15], or removing fungal infections [39]. Previous research on *K. eiffingeri* showed that males guard oviposition sites and use limb or body contact to moisten eggs [30, 31, 40]. We observed that males covered the eggs with their bodies and closely monitored *P. martensi* to protect embryos when the predator was present, and males pushed their forelimbs or covered *P. martensi* with their bodies to expel the predator (personal observation, see video in Additional file 3). These behaviors suggest the importance of paternal care for the survival of embryos.

The males in the experimental group showed a higher attendance frequency than those in the control group, indicating the ability of *K. eiffingeri* males to adjust their egg-guarding behavior when facing an embryo predator. Some frog species have been found to have fixed and high-frequency guarding behaviors (e.g.,* C. hansenae* [15, 41], *Hylophorbus rufescens* [39], and *Hyalinobatrachium valerioi* [42]). However, studies indicate that some frog species can adjust parental care based on environmental factors such as weather conditions [7] and seasons [8]. In previous studies in *K. eiffingeri*, the presence of guarding males reduces embryo predation by *P. martensi* and fungal infections, promoting successful hatching [32]. For male brooding, however, we found that males in the experimental group were only marginally (but not significantly) higher than those in the control group. We contend that this might be because the function of brooding differs from that of attendance. In many glassfrog (Centrolenidae) species, adults moisten eggs by their brooding [16, 18]. In *K. eiffingeri*, previous studies have shown that males might also engage in brooding behavior to maintain egg moistening [30, 31, 40]. Therefore, when faced with the presence of predator, the increase in brooding response in males is not as significant as the increase in attendance. Overall, this study not only reconfirms the importance of paternal care to embryo survival but also demonstrate experimentally the plasticity of paternal care behavior.

### Paternal care strategy: care more in early stage than in late stage due to stage-dependent vulnerability of eggs

The harm to offspring hypothesis proposes that parents provide more care to younger, more vulnerable offspring [21, 26], while the offspring value hypothesis suggests parents invest more care in older offspring as their value is higher [1, 22, 23]. Previous research indicates that parental care during the early developmental stages is critical for the survival of embryos, as embryos in the early stages are more susceptible to drying, predation, or fungal infection, requiring more parental care [43]. Our findings provide direct evidence that embryos in the early developmental stages are more prone to predation. Integrating the evidence of males providing more care for early-stage than late-stage embryos and the higher predation percentage in early-stage embryos, we contend that early-developing embryos under male care experience higher survivorship. Conversely, in the later stages of development, embryos exhibit better defensive capabilities, potentially resulting in reduced parental care of male frogs.

The predation percentage on embryos was higher in the early developmental stages and lower in the later stages, suggesting embryos possess enhanced survival capabilities (resistance to predators) in the later stages. The growth and development of prey often influence the dynamic interactions between prey and predators [44]. For example, in *Oophaga pumilio*, it has been observed that offspring in later developmental stages exhibit some level of toxicity, reducing the risk of predation [45]. In *Agalychnis callidryas*, embryos in the later developmental stages can avoid predation by hatching earlier when attacked by predators [46]. Currently, we do not know whether *K. eiffingeri* possesses chemical defenses against predators. In this study, however, we observed vigorous shaking behavior in late-stage embryos of *K. eiffingeri* in response to *P. martensi*’s stimuli (personal observation). The vigorous shake by late-stage embryos may hinder *P. martensi* from handling and consuming them. Nevertheless, we have observed that late-stage embryos of *K. eiffingeri* showed vigorous shaking responses to various stimuli, such as male care, human manipulation, and light disturbances both in the wild and lab (personal observation). Therefore, further research is needed to determine whether embryos can distinguish between stimuli from predators, threats, or parental care, and whether embryos can possess chemical defenses against predators.

### Reproductive strategy: trade-off

The adjustment of attendance frequency by male *K. eiffingeri* based on the predation pressure and developmental stage of embryos suggests a trade-off between male attendance and costs. Parental care comes with various costs [14], leading parents to weigh the costs and benefits of care based on environmental conditions and adjust the level of care accordingly [23, 41, 47]. In *Allobates femoralis*, adults increase the number of tadpoles transported to minimize energy loss rather than repeat transport if the destination is distant [47]. In *Thoropa taophora*, because egg predators may be more active at night, males tend to guard at night and temporarily abandon egg clutches during the daytime when the risk of dehydration is higher [48]. In *Centrolene savageii*, eggs become less prone to drying as they develop. Consequently, males gradually reduce guarding frequency and display more vocalization behaviors [16]. In these cases, parents evaluate the costs and benefits of guarding based on the current situation, ultimately choosing to alter parental care. In *K. eiffingeri*, a previous study suggests that males have the ability to choose breeding sites based on environmental conditions to maximize offspring survival [49]. Moreover, the authors suggested that males are able to balance between guarding and additional breeding opportunities, adjusting guarding time to maximize reproductive benefits. For example, when water levels inside bamboo stumps are high, reducing the risk of egg dehydration, males should decrease guarding and seek new mating opportunities, and vice versa [31]. Another study also showed that *K. eiffingeri* males adjusted their attending behavior when facing predators that may consume the adult frogs [33]. Our results indicate that when the egg clutch faces a threat from egg predators, males increase attendance frequency to ensure the current offspring’s survival for reproductive benefits. Conversely, when the risk of embryo predation is low, males exhibit lower attendance frequency, suggesting a possible shift towards foraging to replenish energy or seeking new reproductive opportunities, reducing the cost of guarding. Nevertheless, there is currently no direct evidence indicating that male *K. eiffingeri* engaged in parental care suffer costs. These hypothesized costs of parental care, thus, remain to be further studied.

## Conclusion

Previous studies showed an association between offspring age and paternal care but without directly assessing the vulnerability of offspring age on predation risk [21, 27, 28]. Our results provide empirical evidence to support the harm to offspring hypothesis in which male *K. eiffingeri* care more for younger offspring which are more vulnerable. By integrating field and laboratory experiments that include interactions between males, offspring, and offspring predators, we provide direct evidence to explore the plasticity of paternal care in *K. eiffingeri*, which shed light to a better understanding of ecological and evolutionary significance of male reproductive strategies.

### Supplementary Information

Supplementary Material 1.

Supplementary Material 2.

Supplementary Material 3.

## Data Availability

The dataset supporting the conclusions of this article is in an additional file (Additional file 2).

## Abbreviation

SVL: Snout-vent length

## References

[1] Clutton-Brock TH (2019). The evolution of parental care.

[2] de Moraes PZ, Diniz P, Macedo RH. Sex-specific effects of predation risk on parental care in a sexually dichromatic Neotropical songbird. J Avian Biol. 2020;51. 10.1111/jav.02483.

[3] Rangeley RW, Godin J-GJ (1992). The effects of a trade-off between foraging and brood defense on parental behaviour in the convict cichlid dish, *Cichlasoma Nigrofasciatum*. Behaviour.

[4] Smith C, Wootton RJ (1995). The costs of parental care in teleost fishes. Rev Fish Biol Fisheries.

[5] Alonso-Alvarez C, Velando A, Royle NJ, Smiseth PT, Kölliker M (2012). Benefits and costs of parental care. The evolution of parental care.

[6] Gross MR (2005). The evolution of parental care. Q Rev Biol.

[7] Delia JRJ, Ramírez-Bautista A, Summers K (2013). Parents adjust care in response to weather conditions and egg dehydration in a Neotropical glassfrog. Behav Ecol Sociobiol.

[8] Lehtinen RM, Green SE, Pringle JL (2014). Impacts of paternal care and seasonal change on offspring survival: a multiseason experimental study of a Caribbean frog. Ethology.

[9] Carlisle TR (1982). Brood success in variable environments: implications for parental care allocation. Anim Behav.

[10] Ghalambor CK, Martin TE (2002). Comparative manipulation of predation risk in incubating birds reveals variability in the plasticity of responses. Behav Ecol.

[11] Stahlschmidt Z, DeNardo DF (2010). Parental behavior in pythons is responsive to both the hydric and thermal dynamics of the nest. J Exp Biol.

[12] Huang W-S, Lin S-M, Dubey S, Pike DA (2013). Predation drives interpopulation differences in parental care expression. J Anim Ecol.

[13] Crump ML, Rosenblatt JS (1996). Parental care among the amphibia. Advances in the study of behavior.

[14] Machado G, Macedo-Rego RC (2023). Benefits and costs of female and male care in amphibians: a meta-analytical approach. Proc R Soc B Biol Sci.

[15] Poo S, Bickford DP (2013). The adaptive significance of egg attendance in a south-east asian tree frog. Ethology.

[16] Ospina-L AM, Navarro-Salcedo P, Rios-Soto JA, Duarte-Marín S, Vargas-Salinas F (2020). Temporal patterns, benefits, and defensive behaviors associated with male parental care in the glassfrog *Centrolene savagei*. Ethol Ecol Evol.

[17] Gonçalves VF, de Brito-Gitirana L (2008). Structure of the sexually dimorphic gland of *Cycloramphus fuliginosus* (Amphibia, Anura, Cycloramphidae). Micron.

[18] Schulte LM, Ringler E, Rojas B, Stynoski JL (2020). Developments in amphibian parental care research: history, present advances, and future perspectives. Herpetol Monogr.

[19] Valencia-Aguilar A, de Jesus RD, Prado CPA (2020). Male care status influences the risk-taking decisions in a glassfrog. Behav Ecol Sociobiol.

[20] Haase A, Pröhl H (2002). Female activity patterns and aggressiveness in the strawberry poison frog *Dendrobates pumilio* (Anura: Dendrobatidae). Amphibia-Reptilia.

[21] Dale S, Gustavsen R, Slagsvold T (1996). Risk taking during parental care: a test of three hypotheses applied to the pied flycatcher. Behav Ecol Sociobiol.

[22] Trivers RL (2015). Parent-offspring conflict. Am Zool.

[23] Winkler DW (1987). A general model for parental care. Am Nat.

[24] Montgomerie RD, Weatherhead PJ (1988). Risks and rewards of nest defence by parent birds. Q Rev Biol.

[25] RytköNen S, Orell M, Koivula K (1995). Pseudo Concorde fallacy in the willow tit?. Anim Behav.

[26] Swaisgood RR, Rowe MP, Owings DH (2003). Antipredator responses of California ground squirrels to rattlesnakes and rattling sounds: the roles of sex, reproductive parity, and offspring age in assessment and decision-making rules. Behav Ecol Sociobiol.

[27] Goubault M, Scott D, Hardy ICW (2007). The importance of offspring value: maternal defence in parasitoid contests. Anim Behav.

[28] Koskela E, Juutistenaho P, Mappes T, Oksanen TA (2000). Offspring defence in relation to litter size and age: experiment in the bank vole *Clethrionomys glareolus*. Evol Ecol.

[29] Kam Y-C, Chuang Z-S, Yen C-F (1996). Reproduction, oviposition-site selection, and tadpole oophagy of an arboreal nester, *Chirixalus eiffingeri* (Rhacophoridae), from Taiwan. J Herpetol.

[30] Chen Y-H, Yu H-T, Kam Y-C (2007). The ecology of male egg attendance in an arboreal breeding frog, *Chirixalus eiffingeri* (Anura: Rhacophoridae), from Taiwan. Zoolog Sci.

[31] Cheng W-C, Kam Y-C (2010). Paternal care and egg survivorship in a low nest-attendance rhacophorid frog. Zool Stud.

[32] Chuang M-F, Borzée A, Kam Y-C (2019). Attendance to egg clutches by male *Kurixalus eiffingeri* increases hatching success and decreases predation by invasive slugs (*Parmarion martensi*) in Taiwan. Ethology.

[33] Chuang M-F, Lee W-H, Sun J-S, You C-H, Kam Y-C, Poo S (2017). Predation risk and breeding site value determine male behavior and indirectly affect survivorship of their offspring. Behav Ecol Sociobiol.

[34] Phillott AD, Skerratt LF, McDonald KR, Lemckert FL, Hines HB, Clarke JM, Alford RA, Speare R (2007). Toe-clipping as an acceptable method of identifying individual anurans in mark recapture studies. Herpetol Rev.

[35] Donnelly MA, Guyer C, Juterbock EJ, Alford RA, Heyer W, Donnelley M, McDiarmid R, Hayek L, Foster M (1994). Techniques for marking amphibians. Measuring and monitoring biological diversity: standard methods for amphibians.

[36] Poo S, Cheng Y-C, Fuh N-T, Chuang M-F, Kam Y-C. Sex-specific oviposition site selection in an arboreal treefrog with a resource-defense mating system. Ethology. 2024:e13444. 10.1111/eth.13444.

[37] Gosner KL (1960). A simplified table for staging anuran embryos and larvae with notes on identification. Herpetologica.

[38] Hughey MC, Delia J, Belden LK (2017). Diversity and stability of egg-bacterial assemblages: the role of paternal care in the glassfrog *Hyalinobatrachium colymbiphyllum*. Biotropica.

[39] Bickford DP (2004). Differential parental care behaviors of arboreal and terrestrial microhylid frogs from Papua New Guinea. Behav Ecol Sociobiol.

[40] Ueda H (1986). Reproduction of *Chirixalus eiffingeri* (Boettger). Sci Rep Lab Amphibian Biol.

[41] Poo S, Evans TA, Tan MK, Bickford DP (2016). Dynamic switching in predator attack and maternal defence of prey. Biol J Lin Soc.

[42] Vockenhuber EA, Hödl W, Karpfen U (2008). Reproductive behaviour of the glass frog *Hyalinobatrachium valerioi* (Anura: Centrolenidae) at the tropical stream Quebrada Negra (La Gamba, Costa Rica). Stapfia.

[43] Townsend DS, Stewart MM, Pough FH (1984). Male parental care and its adaptive significance in a neotropical frog. Anim Behav.

[44] Polis GA (1984). Age structure component of niche width and intraspecific resource partitioning: can age groups function as ecological species?. Am Nat.

[45] Stynoski JL, Shelton G, Stynoski P (2014). Maternally derived chemical defences are an effective deterrent against some predators of poison frog tadpoles *Oophaga pumilio*. Biol Let.

[46] Warkentin KM (2000). Wasp predation and wasp-induced hatching of red-eyed treefrog eggs. Anim Behav.

[47] Ringler E, Pašukonis A, Hödl W, Ringler M (2013). Tadpole transport logistics in a Neotropical poison frog: indications for strategic planning and adaptive plasticity in anuran parental care. Front Zool.

[48] Consolmagno RC, Requena GS, Machado G, Brasileiro CA (2016). Costs and benefits of temporary egg desertion in a rocky shore frog with male-only care. Behav Ecol Sociobiol.

[49] Lin Y-S, Lehtinen RM, Kam Y-C (2008). Time- and context-dependent oviposition site selection of a phytotelm-breeding frog in relation to habitat characteristics and conspecific cues. Herpetologica.

