# Supplementary material for: Paternal care plasticity: males care more for early- than late-developing embryos in an arboreal breeding treefrog
Source: Front Zool. 2024 Jun 19;21:16. doi: 10.1186/s12983-024-00537-z (PMC11186214; doi:10.1186/s12983-024-00537-z)
Supplement: Supplementary file 1 — Supplementary Material 1. [file 12983_2024_537_MOESM1_ESM.docx]

**Supplementary Tables**

**Table S1. Spearman correlations evaluating the influence of male body size (SVL) and bamboo stump on daily egg mortality**

| characteristics | *r*_s_ | *P* |
| --- | --- | --- |
| Male SVL (mm) | -0.08 | 0.674 |
| Stump height (cm) | -0.27 | 0.141 |
| Stump diameter (mm) | 0.12 | 0.503 |
| Stump depth (cm) | -0.05 | 0.780 |
| Water depth (cm) | 0.03 | 0.862 |

**Table S2. Comparison of** **male body size (SVL) and bamboo stump characteristics between control and experimental groups**

| characteristics | Control groups (n=8) | | Experimental groups (n=7) | | Wilcoxon signed-rank test | |
| --- | --- | --- | --- | --- | --- | --- |
|  | Median | Min-Max | Median | Min-Max | *Z*-value | *P*-value |
| Male SVL (mm) | 25.7 | 23.2-29.5 | 26.9 | 23.4-34.4 | 0.98 | 0.325 |
| Stump height (cm) | 57.0 | 16.9-106.6 | 46.8 | 31.3-127.6 | -0.17 | 0.862 |
| Stump diameter (mm) | 71.7 | 58.5-92.2 | 74.8 | 60.9-83.7 | 0.06 | 0.954 |
| Stump depth (cm) | 10.0 | 7.0-17.3 | 11.1 | 6.9-25.3 | 0.64 | 0.524 |
| Water depth (cm) | 6.9 | 2.5-10.8 | 6.8 | 4.8-17.8 | 0.58 | 0.563 |

**Table S3. Spearman correlations evaluating the influence of the body weight of *P. martensi* on the percentage of embryo predation**

|  | Overall (early stage+ late stage, n = 40) | |  | Late stage (n = 20) | |
| --- | --- | --- | --- | --- | --- |
| Variable | *r*_s_ | *P* |  | *r*_s_ | *P* |
| Body weight (g) | -0.11 | 0.488 |  | -0.03 | 0.895 |

The analyses were performed separately for the relationships between *P. martensi*'s weight and the overall percentage of embryo predation (early stage + late stage) and between *P. martensi*'s weight and the percentage of late-stage embryo predation. Since the percentage of embryo predation in the early stage was 100% for all cases, no analysis was performed for this group.
